# Acute Lung Injury in Cholinergic-Deficient Mice Supports Anti-Inflammatory Role of α7 Nicotinic Acetylcholine Receptor

**DOI:** 10.3390/ijms22147552

**Published:** 2021-07-14

**Authors:** Nathalia M. Pinheiro, Rosana Banzato, Iolanda Tibério, Marco A. M. Prado, Vânia F. Prado, Ayman K. Hamouda, Carla M. Prado

**Affiliations:** 1 Department of Bioscience, Federal University of Sao Paulo, Santos 11015-020, SP, Brazil; pinheiro.nathalia@gmail.com; 2 College of Pharmacy, University of Texas at Tyler, Tyler, TX 75799, USA; AHamouda@uttyler.edu; 3 Department of Medicine, School of Medicine, University of Sao Paulo, Sao Paulo 01246-903, SP, Brazil; rbanzato.rb@gmail.com (R.B.); iocalvo@uol.com.br (I.T.); vprado@robarts.ca (V.F.P.); 4 Molecular Medicine Group, Robarts Research Institute, London, ON N6A 5B7, Canada; mprado@robarts.ca; 5 Department of Physiology & Pharmacology, University of Western Ontario, London, ON N6A 5B7, Canada; 6 Department of Anatomy & Cell Biology, University of Western Ontario, London, ON N6A 5B7, Canada

**Keywords:** nicotinic acetylcholine receptors, muscarinic acetylcholine receptors, acute lung injury, PNU 282987, cholinergic anti-inflammatory pathway

## Abstract

(1) Background: The lung cholinergic pathway is important for controlling pulmonary inflammation in acute lung injury, a condition that is characterized by a sudden onset and intense inflammation. This study investigated changes in the expression levels of nicotinic and muscarinic acetylcholine receptors (nAChR and mAChR) in the lung during acute lung injury. (2) Methods: acute lung injury (ALI) was induced in wild-type and cholinergic-deficient (VAChT-KD^HOM^) mice using intratracheal lipopolysaccharide (LPS) instillation with or without concurrent treatment with nicotinic ligands. Bronchoalveolar lavage fluid was collected to evaluate markers of inflammation, and then the lung was removed and processed for isolation of membrane fraction and determination of acetylcholine receptors level using radioligand binding assays. (3) Results: LPS-induced increase in lung inflammatory markers (e.g., neutrophils and IL-1β) was significantly higher in VAChT-KD^HOM^ than wild-type mice. In contrast, LPS treatment resulted in a significant increase in lung’s α7 nicotinic receptor level in wild-type, but not in VAChT-KD^HOM^ mice. However, treatment with PNU 282987, a selective α7 nicotinic receptor agonist, restored VAChT-KD^HOM^ mice’s ability to increase α7 nicotinic receptor levels in response to LPS-induced acute lung injury and reduced lung inflammation. LPS also increased muscarinic receptors level in VAChT-KD^HOM^ mice, and PNU 282987 treatment reduced this response. (4) Conclusions: Our data indicate that the anti-inflammatory effects of the lung cholinergic system involve an increase in the level of α7 nicotinic receptors. Pharmacological agents that increase the expression or the function of lung α7 nicotinic receptors have potential clinical uses for treating acute lung injury.

## 1. Introduction

Acute respiratory distress syndrome (ARDS) is defined as a diffuse and acute lung inflammatory, and characterized by the presence of alveolar edema with reduced pulmonary complacency and a reduction in the relationship between partial oxygen pressure and an oxygen inspired fraction [1]. The mortality rate for this syndrome still varies between 30–50% [2,3,4]. Despite the high mortality, ARDS does not have a specific treatment, and the main aim of the therapy is to treat the underlying cause of the disease and provide adequate ventilator support to maintain the patient’s oxygenation [2,4]. Lung inflammatory response includes an increase in the permeability of the alveolar-capillary barrier, leading to pulmonary edema and enhanced disposition of collagen fibers in the extracellular matrix [5,6]. In addition, elevated levels of IL-8, an essential chemotactic factor for neutrophils, were found in patients with ARDS [7].

The cholinergic anti-inflammatory system was initially identified as a neural mechanism that suppresses the innate inflammatory response and controls inflammation by inhibiting the release of pro-inflammatory cytokines such as TNF-α [8]. The detection of all components of the cholinergic system, including high-affinity choline transporter, vesicular acetylcholine transporter (VAChT), and choline acetyltransferase in the lung and immune cells, suggests a possible role of the cholinergic system in the pathophysiology of respiratory diseases [9]. Acetylcholine (ACh), mainly released via the vagus nerve, acts on muscarinic acetylcholine receptors (mAChRs), which are G-protein coupled receptors, and nicotinic acetylcholine receptors (nAChRs), which are ligand-gated ion channels. Within the respiratory system, mAChRs are finding n smooth muscles, inflammatory cells, and epithelial cells, and their physiological role in the lung is bronchoconstriction, bronchial secretion, and ciliary beat [10,11]. The α7 nAChR represents the major nAChRs within the respiratory system expressed in macrophages and other non-neuronal cells [12]. Animals with deletion of the *α7 nAChR* gene exhibit increased LPS-induced TNF-α release, and this response was not reversed upon vagal stimulation, suggesting a vital role of this receptor in controlling the anti-inflammatory response [13]. Moreover, the use of α7nAChR agonists reduced inflammatory cells and the pulmonary release cytokines in acute lung injury (ALI) models [12,14,15]. We previously showed that animals with ALI treated with nAChR agonist PNU 282987 significantly reduced lung inflammatory responses due to a change in the macrophages profile from M1 to M2-like [16].

The role of ACh in lung diseases is still debatable, given that cholinergic activation can perpetuate or suppress the inflammatory response depending on the cholinergic receptor that ACh acts on the lung [17]. Nevertheless, the anti-inflammatory properties of agonist and allosteric modulation of nAChR have been well studied [18,19]. Thus, studies that clarify the cholinergic anti-inflammatory pathway mechanisms are significant for developing new therapeutic strategies targeting the lung cholinergic receptors. However, few studies have focused on the function and expression of various nicotinic receptor subtypes during ALI. This study uses lipopolysaccharides (LPS)-induced ALI model in WT and cholinergic-deficient (VAChT-KD^HOM^) mice with or without concurrent treatment with nicotinic ligands to investigate changes in the expression levels of nAChRs and mAChRs in the lung during ALI.

The intratracheal instillation of LPS increased peripheral blood neutrophils and the number of neutrophils recruited to the lung after 6 h. There is pulmonary remodeling after LPS and damage in lung functions similar to those observed in the ARDS physiopathology [16,20]. VAChT-KD^HOM^ mutant mice were generated as described by Prado, Martins-Silva et al. (2006); they were knock-down to the *VAChT* gene and showed a 65% reduction in VAChT level. The content of ACh released in the synaptic cleft is directly proportional to the amount of VAChT [21,22,23], which is why VAChT-KD^HOM^ mice have been used to study cholinergic deficiency [24]. Our group demonstrated that VAChT reduction in animals with no pathological stimulus in the lung, such as LPS or ovalbumin, caused inflammatory cell recruitment to the lung, increased pro-inflammatory cytokines release, activation of nuclear factor kappa B (NF-kB), and reduction in the activation of the Janus kinase—signal transducer and activation of the transcription—suppressor of cytokine signaling (JAK2-STAT3-SOCS3) pathway. This inflammatory process induced an increase in extracellular matrix fiber deposition, suggesting that the lung is in the process of remodeling [17].

Here, we report that LPS-induced increase in lung inflammatory markers was significantly higher, and α7 nAChR level was lower, in VAChT-KD^HOM^ compared to WT mice, consistent with the protective anti-inflammatory role of lung cholinergic system that involves the α7 nAChR. Furthermore, treatment with PNU 282987 which acts as a highly selective α7 nAChR agonist [25,26], restored VAChT-KD^HOM^ mice ability to increase α7 nAChR levels and reduce lung inflammation. Altogether, this data supports the potential therapeutic benefit of pharmacological agents that increase the expression and/or the function of lung α7 nicotinic for patients who develop ALI.

## 2. Results

**Figure 1:**
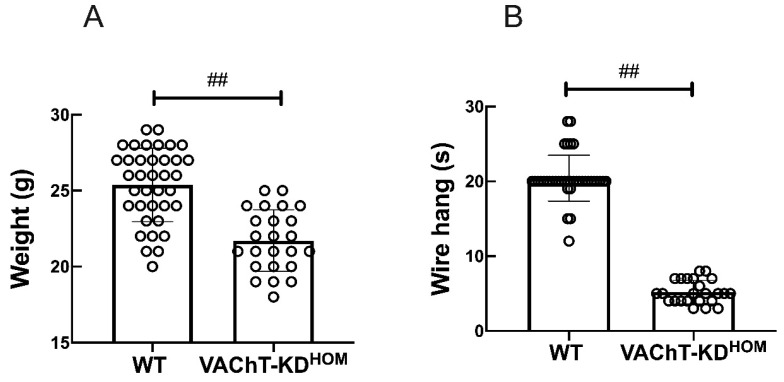
Comparison of body weights and motor functions of VAChT-KD^HOM^ and WT mice. (**A**) Mice body weight in gram and the latency (time in seconds) that mice spent before fall in the wire hang test (**B**) were recorded before LPS instillation or PNU treatment. Data are expressed as mean ± SD of 37 mice in the WT group and 24 mice in the VAChT-KD^HOM^. ## indicates a statistically significant difference *p* < 0.001. The value obtained from each animal is represented by a circle.

To study the role of the lung cholinergic system in ALI, we employ a previously established cholinergic deficient VAChT homozygous (VAChT-KD^HOM^) mutant mice [23]. VAChT-KD^HOM^ mice had a 65% reduction in VAChT level in the central nervous system and in the lung [17,23], and phenotypically characterized by significant neuromuscular deficits and impaired object and social recognition skills. Figure 1A shows body weight measurements and performance in the wire-hang test for VAChT-KD^HOM^ and WT mice used in studies reported in this report. As previously described [17], VAChT-KD^HOM^ mice had lower body weights (Figure 1A) and were myasthenic, unable to hold their weight, and performed poorly in the wire-hang test (Figure 1B) in comparison to WT mice.

### 2.1. Effect of VAChT Deficiency and PNU 282987 Treatment on LPS-Induced Lung Inflammation

**Figure 2:**
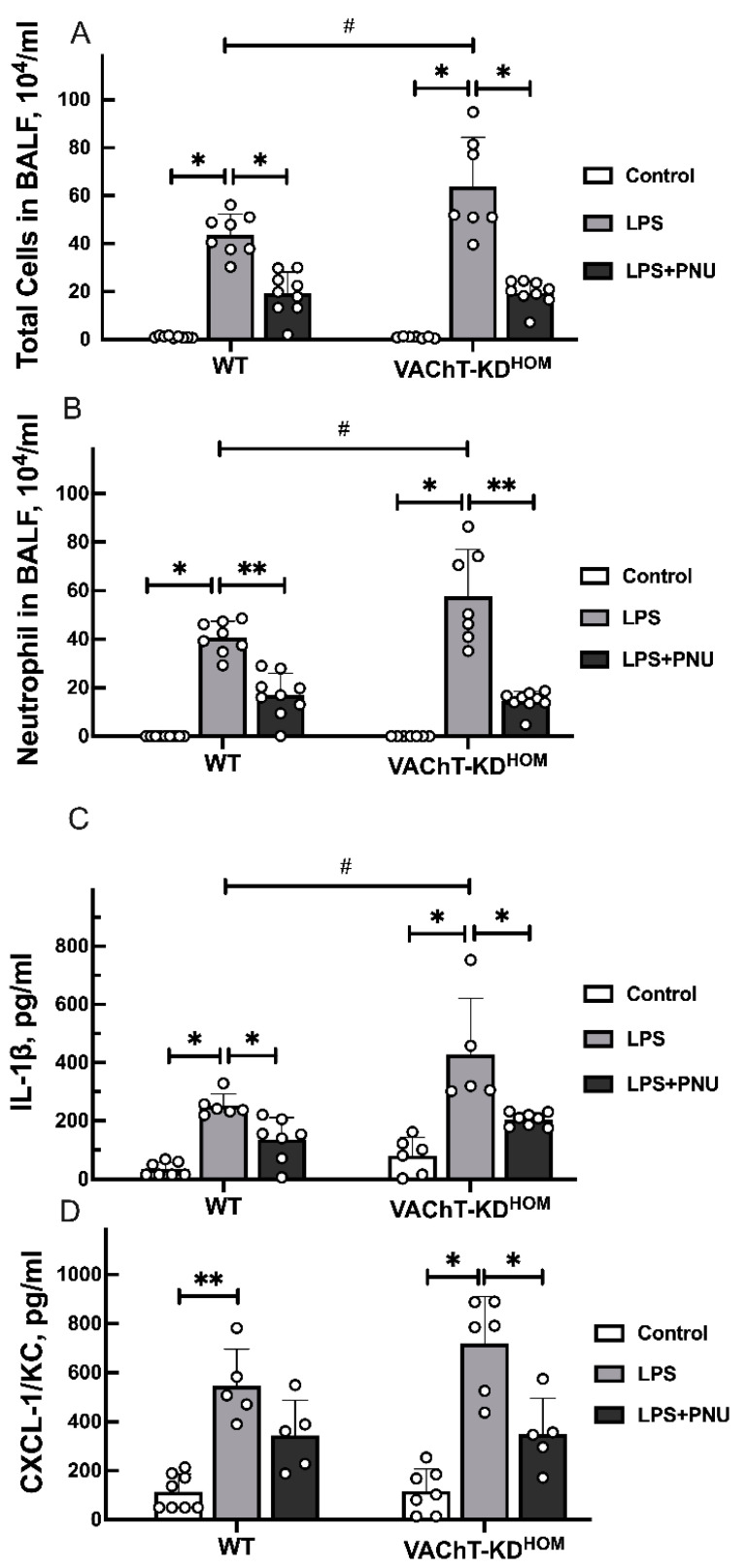
Levels of inflammatory markers in BALF of WT and VAChT-KD^HOM^ mice. (**A**) Total cells; (**B**) Neutrophil; (**C**) IL-1β; and (**D**) CXCL-1/KC were measured in BALF of WT and VAChT-KD^HOM^ collected 24 h following intratracheal LPS instillation with (LPS+PNU groups) or without (LPS groups) treatment with PNU 282987 and compared to LPS-untreated WT and VAChT-KD^HOM^ mice (control groups) as described in Section 4.5 and Section 4.6. Data are expressed as mean ± SD of 5–9 mice per group. Significant differences are based on Holm–Šidak post hoc test following one-way ANOVA. Statistically significant differences versus LPS-untreated control group with the same genetic background are indicated with asterisks (** *p* < 0.01 and * *p* < 0.05), and significant differences between WT and VAChT-KD^HOM^ groups receiving the same experimental treatment are indicated in hashtags (# *p* < 0.05). The value obtained from each animal is represented by a circle.

To evaluate ALI severity following LPS instillation in WT and VAChT-KD^HOM^ with or without treatment with PNU 282987, BALF was retrieved, and the levels of lungs inflammation markers were determined (Figure 2).

LPS instillation resulted in a significant increase in total cells (Figure 2A), neutrophils (Figure 2B), Interleukin 1 beta (IL-1β) (Figure 2C), and chemokine ligand 1 (CXCL-1/KC) cytokine levels (Figure 2D) levels in BALF of WT and VAChT-KD^HOM^ mice. In addition, LPS-induced increase in total cells, neutrophils, and IL-1β was significantly higher in VAChT-KD^HOM^ mice than in WT mice. Treatment with PNU 282987 significantly reduced LPS-induced increase in the levels of total cells, neutrophils, and IL-1β in WT and VAChT-KD^HOM^ LPS animals. PNU 282987 treatment also reduced LPS-induced increase in CXCL-1/KC in WT and VAChT-KD^HOM^ mice compared to their PNU 282987-untreated counterparts, although this effect was only statistically significant for the VAChT-KD^HOM^ mice.

### 2.2. Effect of VAChT Deficiency, LPS-Instillation, and PNU 282987 Treatment on the Level of Lung Cholinergic Receptors

**Figure 3:**
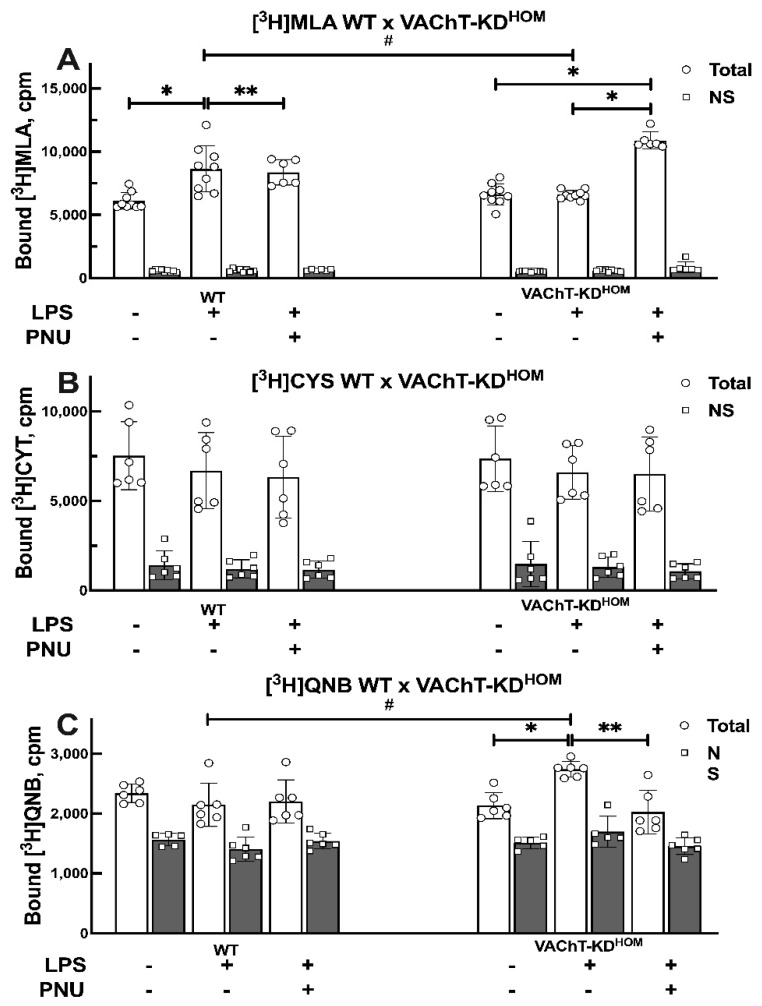
Quantification of lung nAChRs and mAChRs in WT and VAChT-KD^HOM^ mice. Membrane fractions were isolated from lungs of WT and VAChT-KD^HOM^ mice, collected 24 h following intratracheal LPS instillation with (LPS + PNU groups) or without (LPS groups) treatment with PNU 282987, and compared to LPS-untreated WT and VAChT-KD^HOM^ mice (control groups) a described in Section 4.7.1 The level of homomeric α7 nAChR, heteromeric nAChR, and mAChRs were determined using the specific binding of [^3^H] MLA (**A**), [^3^H] Cytisine (**B**), and [^3^H]QNB (**C**), respectively. Data for [^3^H] MLA (**A**), [^3^H] Cytisine (**B**), and [^3^H]QNB (**C**) are mean ± SD of 2–3 independent experiments, each conducted in triplicate as described in Section 4.7.2 Significant differences are based on Holm–Šidák post hoc test following one-way ANOVA. Statistically significant differences versus LPS-untreated control group with same genetic background are shown with asterisks (** *p* < 0.01 and * *p* < 0.05), and significant differences between WT and VAChT-KD^HOM^ groups receiving the same experimental treatment are shown in hashtags (# *p* < 0.05). The value obtained from each animal is represented by a circle.

We used radioligand binding assay for quantification of major nAChRs and mAChRs in membrane fractions isolated from the lung from control LPS-untreated WT and VAChT-KD^HOM^ mice, and WT and VAChT-KD^HOM^ mice following LPS instillation with or without treatment with PNU 282987 (Figure 3). The specific binding of [^3^H]Methyllycaconitine citrate ([^3^H]MLA), a nAChR antagonist with higher selectivity at the homomeric α7 than heteromeric nAChR [27,28,29], was used for quantification of α7 nAChR in lung membrane fractions (Figure 3A). The specific binding of [^3^H]Cytisine, a partial nAChR agonist that binds preferentially to the β2-containing nAChR subtype [30,31], was used to quantify heteromeric nAChRs (e.g., α4β2) in lung membrane fractions (Figure 3B). The specific binding of [^3^H]-Quinuclidinyl benzilate ([^3^H]QNB), a widely used specific mAChRs antagonist [32,33,34,35], was used to quantify mAChR in lung membrane fractions (Figure 3C). There was no significant change in the level of nAChRs or mAChRs in lungs isolated from LPS-untreated control VAChT-KD^HOM^ mice compared to LPS-untreated control WT mice. However, LPS instillation resulted in an increase in the α7 nAChR levels in the lungs of WT mice, but not in the VAChT-KD^HOM^ mice (Figure 3A). Treatment of VAChT-KD^HOM^ mice with PNU 282987 reduced LPS-induced increase in the lung α7 nAChR levels. Unlike α7 nAChR, the level of heteromeric nAChRs in the lung was not affected by LPS-instillation or PNU 282987 treatment in WT or VAChT-KD^HOM^ mice (Figure 3B). LPS instillation increased lung mAChR level in the VAChT-KD^HOM^ mice, but not in the WT mice (Figure 3C). Treatment with PNU 282987 abolished LPS-induced increase in lung mAChRs in VAChT-KD^HOM^ mice, and had no effect on lung mAChR in WT mice.

### 2.3. Effect of Vagotomy in LPS-Induced Lung Inflammation and Cholinergic Receptors in WT Mice

**Figure 4:**
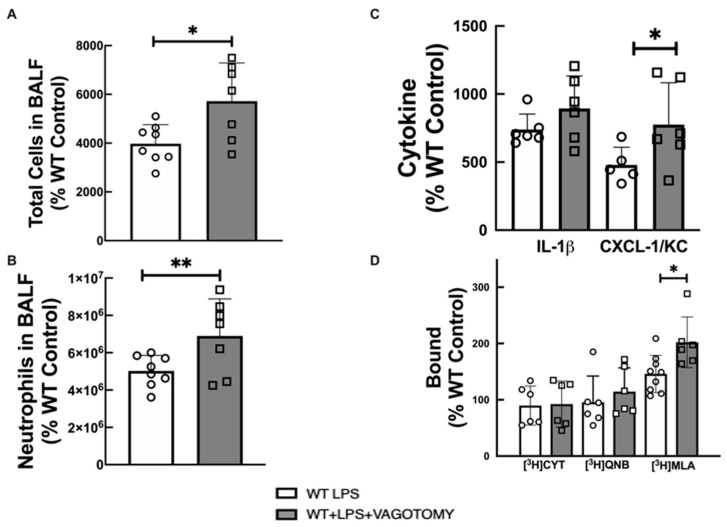
Effect of vagotomy on lung inflammatory markers and cholinergic receptors. (**A**) total cells in BALF, (**B**) neutrophils in BALF, (**C**) Cytokine in BALF, and (**D**) [^3^H]cytisine, [^3^H]QNB, and [^3^H]MLA binding to lung membrane fractions of LPS-treated WT. Statistically significant effects of vagotomy based on one-way ANOVA versus LPS-treated WT group are shown in asterisks (** *p* < 0.01, and * *p* < 0.05). Data are expressed as mean ± SD of 6–8 mice per group. The value obtained from each animal is represented by a circle.

Vagotomy (removal of vagus nerve) is known to acutely reduce cholinergic tone [36]. Vagotomy following LPS-instillation resulted in increased lung inflammatory markers with a statistically significant increase in total cells (Figure 4A), neutrophils (Figure 4B), and CXCL-1/KC (Figure 4C) in BALF, and increased lung α7 nAChR levels (Figure 4D) when compared to LPS-treated WT mice. Vagotomy did not significantly alter IL-1β level in BALF, or lung level of heteromeric nAChRs and mAChRs.

## 3. Discussion

The present study demonstrated that the deficiency of VAChT increased lung inflammation in animals that received LPS instillation, supporting the notion of the importance of endogenous ACh in the control of inflammation in the respiratory system. These results are also consistent with the vagotomy results, which present similar, albeit not identical, receptor and inflammatory changes. The reduction in VAChT levels induced upregulation in inflammatory cells, particularly neutrophils and IL-1β, and increased muscarinic receptors in the lung after LPS instillation. Moreover, the increase in α7 nAChR in the lung in response to LPS instillation in WT mice suggests a tentative regulation of lung inflammation, and its absence in VAChT-KD^HOM^ animals is coincident with more pronounced lung inflammation. These data suggest the anti-inflammatory cholinergic system is involved in the modulation of pulmonary inflammation, and that the stimulation of nicotinic receptors can be an attractive therapeutic target to be explored in ALI and other inflammatory lung diseases.

VAChT-KD^HOM^ mice have a reduction of approximately 65% in the release of ACh due to genetic reduction in VAChT [21,23]. The decrease in ACh release induces a reduction in body weight and the animals maintenance time in the wire-hang test that corroborates previous results that VAChT-KD^HOM^ animals have myasthenia and impaired neuromuscular function [23,37].

Considering that acute respiratory distress syndrome has a high mortality, evaluating the role of cholinergic deficiency in an ALI model would be of great value in reveal mechanisms and therapeutic targets. Lips et al. (2007) [38] demonstrated a reduction in cholinergic components, including VAChT, in a model of acute allergic airway inflammation. Furthermore, Wessler et al. (2007) [39] observed a significant reduction in the ACh content in the airways and the pulmonary parenchyma of patients with cystic fibrosis compared to controls. ALI occurs due to systemic infection and/or a lung injury, and is characterized by the intensive production of inflammatory mediators, called cytokines, by cells of the immune system. It is well known that intratracheal LPS instillation can induce ALI, with intense lung parenchyma inflammation in histological sections and increased edema evaluated by wet/dry lung weight and by total protein, as previously shown [16,20].

VAChT deficient animals have a more pronounced increase in total cells and neutrophils recovered in the BALF and the levels of IL-1β. It was shown that animals with VAChT reduction have a more pronounced lung inflammatory response to diesel exhaust particles [40] and an asthma model [24]. Considering ALI, Santana et al. (2021) [41] demonstrated that cytokine dysfunction observed in female VAChT-KD^HOM^ mice increased the mortality in an ischemia and reperfusion model. Therefore, we showed endogenous cholinergic dysfunction is involved in the development of ALI induced by LPS instillation.

Given the importance of cholinergic receptors to regulate inflammatory responses, we analyzed whether inflammation could regulate receptor levels and how receptors respond to pharmacological treatment with PNU 282987. Mice treated with PNU 282987 showed reduced total cells, neutrophils, and IL-1β, although CXCL-1/KC levels were reduced only in VAChT-KD^HOM^ mice by PNU 28297. These data showed that the α7 nAChR stimulation protects and prevents lung inflammation. Pinheiro et al. (2017) [16] have previously observed, using LPS in C57BL/6 mice, that the PNU 282987 treatment reduced inflammatory cells and cytokine released, lung wet/dry ratio, and M1-like macrophages, whereas this treatment increased M2-like macrophages. Zhao et al. (2019) [15] showed α7 nAChR activation reduced IL-6, TNF-α, and IL-1β LPS-induced in vitro. However, the role of nicotinic receptor levels in the status of cholinergic deficiency is unknown.

To show whether ALI induces alterations in α7 nAChR, we evaluated the levels of these receptors in the lung. LPS upregulated α7 nAChR levels in WT mice, but not in the VAChT-KD^HOM^ LPS, suggesting that this up-regulation may involve the regulation of cholinergic tone that may help to prevent LPS-induced damage in WT animals compared to VAChT-KD^HOM^ mice. However, treatment with PNU 282987, which reduced lung inflammation in VAChT-KD^HOM^ animals, induced an increase in α7 nAChR levels in the VAChT-KD^HOM^+LPS+PNU group. We hypothesized that animals with a long-term cholinergic deficiency could not increase nicotinic receptor levels to counteract lung inflammation, as occurred in WT. In addition, the pharmacological stimulus to the nicotinic receptor by PNU, which decreased lung inflammation in cholinergic deficiency mice, induced increased nicotinic receptors α7. The lack of ACh available for α7 nAChR receptors can modulate pulmonary inflammation due to inhibition of the cholinergic anti-inflammatory pathway [42].

Another type of nicotinic receptor is the α4 nAChR, which is found in the epithelium. However, unlike the α7 nAChR, we did not find any changes in the levels of heteromeric nAChR (predominantly α4β2 nAChR) in response to the LPS insult, nor to the stimulation with PNU 282987.

Muscarinic receptors are very present in the respiratory system and important in reducing inflammation, having a more pro-inflammatory role [43]. To evaluate the levels of mAChR, we were used similar binding assays with a muscarinic ligand. We found that muscarinic receptors were not altered with the reduction in VAChT per se. However, LPS-induced higher increase in mAChR in VAChT-KD^HOM^ mice. Interestingly, the treatment with PNU 282987 reduced the muscarinic receptor expression on the lung from VAChT-KD^HOM^. This effect occurred in VAChT deficiency mice, but not in the WT LPS mice compared to the control group. Verbout et al. (2007) [44] showed that atropine treatment increased airway hyperreactivity induced by the asthma model, and this effect was dependent on inflammation. Additionally, blocking the M3 receptor prevented the increase in TNF-α and MCP-1 associated with inflammatory cell infiltration and tissue damage in a sepsis model [45]. Other authors have also shown evidence that muscarinic receptors can be involved in acute lung inflammation [14,46,47,48]. Taken together, these data suggest that WT animals, in response to LPS insult, respond by increasing the number of nicotinic receptors and do not alter the number of muscarinic receptors, thus have a milder inflammation when compared to VAChT-KD^HOM^ animals. On the other hand, VAChT-KD^HOM^ did not respond to LPS instillation by altering nicotinic receptors. Additionally, they increased muscarinic receptors, perhaps contributing to the increased inflammation observed in these animals.

It is relevant to highlight that we evaluated α7 nAChR, α4 nAChR, and mAChR, and none of the components are altered in the mutant mice [17,49] and, therefore, the effects observed in the lungs of animals with cholinergic deficiency are attributed to the reduction in VAChT, which is associated to a long-term reduction in cholinergic tone in response to an inflammatory insult.

Finally, we evaluated vagotomized WT animals that received LPS instillation, to analyze whether acute cholinergic reduction affects inflammation. Vagotomized mice showed increased total cells, neutrophils, IL-1 β, and CXCL-1/KC in BALF induced by LPS instillation compared to the WT-Control group, similar to VAChT-deficient mice. Other studies have shown that vagotomized animals exhibited increased inflammatory cells after septic peritonitis [50,51]. It is important to note that vagotomy and interfering with the release of ACh may also affect the secretion of peptides and other neurotransmitters [52]. Hofer et al. (2008) [53] evaluated the effect of physostigmine, an AChE inhibitor, and observed that treatment with this drug reduced mortality and pro-inflammatory cytokines TNF-α, IL-1β, and IL-6 in a sepsis model. In the lung [36], it was demonstrated that vagotomy increased lung inflammation, and the vagus nerve stimulation reduced the inflammation induced by LPS instillation. In addition, vagotomy, which acutely reduces cholinergic tone, increased α7 nAChR levels in lung tissue in response to LPS when compared to WT LPS, which differed from the responses observed in VAChT-KD^HOM^ mice. Unlike α7 nAChR, α4 nAChR and mAChR expression were unchanged in the vagotomy group.

In summary, we showed that cholinergic deficiency mice were not able to increase nicotinic receptors in response to LPS instillation, and instead increased the muscarinic receptors, which is pro-inflammatory in the lung. Collectively, these data suggest that cholinergic tone modulates nicotinic and muscarinic receptors, and that nicotinic receptors are essential to counteract lung inflammation in a model of ALI, as pharmacological activation of α7 nAChR in VAChT-deficiency mice reduced lung inflammation while increasing α7 nAChR expression and reducing mAChR. Considering that the use of agonist nicotinic receptors has significant clinical potential, this proposal has excellent relevance for patients who develop ARDS.

## 4. Materials and Methods

Lungs were removed from genetically modified male mice with reduced levels of VAChT. VAChT-KD^HOM^ animals present a reduction of approximately 65% in the VAChT protein, which leads to a reduction in the release of ACh in the same proportion in the central nervous system [21] and the lung [17]. This mice model has been extensively used to evaluate cholinergic deficiency in several diseases. [17,24,37,40,41,54,55]. These animals were taken from the Central Vivarium of the Faculty of Medicine of the University of São Paulo, with an average weight of 20 g, approximately 6–8 weeks old. The animals were kept in environments with controlled temperature (21 to 23 °C), humidity, and a 12-h light/dark cycle, and free access to water and food, following the guidelines established by Brazil National Council for the Control of Animal Experimentation (CONCEA). The study was conducted according to the guidelines of the Declaration of Helsinki, and approved by Ethics Committee of Hospital das Clinicas, Faculty of Medicine, University of São Paulo (protocol code n° 1183/2018 and 22 November 2018).

### 4.1. Weight and Wire-Hang Test

First, the animals were weighed, and the weight was expressed in g. After, WT and VAChT-KD^HOM^ were submitted to the wire-hang test. This involves mice being placed on the cage top lid, and slowly inverting the lid for a maximum time of 60 s. The time upside down was measured [17], and results are shown in seconds.

### 4.2. LPS Instillation Protocol

The animals were anesthetized with inhaled isoflurane (1 mL/3 LO_2_/5%), and an aperture was performed to expose the trachea and intrathecal LPS instillation (Serotype 026: B6/L3755, Sigma Aldrich, St. Louis, MO, USA) diluted in saline at the dose of 1 mg/kg or saline using a needle (caliber 0.38 × 13 mm/BD Plastipak). Under the effect of anesthesia, the animals were sutured with a surgical line (nylon 4.0, NPA373, Brasuture, São Sebastião da Grama, Brazil). Control animals received saline in equal volume. The lungs of the animals were removed 24 after the instillation of LPS.

### 4.3. Treatment with PNU 282987

The groups VAChT-KD^HOM^ + LPS + PNU and WT + LPS + PNU received PNU 282987 treatment (Tocris, Bristol, UK), intraperitoneal, an agonist the α7 nAChR, in the dose of 10 mg/kg per animal, six hours after LPS instillation [16]

### 4.4. Vagotomy

The group (WT + VAG + LPS) was anesthetized with inhaled isoflurane (1 mL/3 LO_2_/5%), and an aperture was performed to expose the vagus nerves that were sectioned. These animals received 1 mg/kg 30 min after the vagotomy surgery.

### 4.5. Bronchoalveolar Lavage Fluid (BALF)

The trachea was cannulated and the BALF was obtained by washing the airways with 3 × 0.5 mL of sterile saline solution. For total and differential cell counts, the BALF was centrifuged at 112.03× *g* for 10 min, and the cell pellet was resuspended in 300 μL of sterile saline. The total number of viable cells was determined in a Neubauer hemocytometer counting chamber. Differential cell counts were performed on BALF cytocentrifuge preparations (450 rpm for 6 min) (Cytospin, Cheshire, UK) stained with Diff-Quick (Biochemical Sciences Inc., Swedesboro, NJ, USA). At least 300 cells were counted according to standard morphological criteria [16].

### 4.6. Immunoenzymatic Assay (ELISA) for Cytokine Detection in BALF

The ELISA was performed on pulmonary homogenate for detection of the levels of IL-1β, CKCL-1/KC cytokines using the Duo-Set kit for mice (R&D Systems, Minneapolis, MN, USA) following the instructions from the manufacturer. For the cytokine’s quantification, the spectrophotometer (Epoch—Bioteck) and the GEN 5.1.1.1 program were used (450 nm). The values were expressed as pg/mL [16].

### 4.7. Evaluation of nAChR Nicotinic Receptors and Identification of Receptor Subunits in Lung Tissue

#### 4.7.1. Preparation of Lung Membrane Fractions

The lung tissue was homogenized in sucrose buffer (0.32 M), vesicle dialysis buffer (VDB) (100 mM NaCl, 0.1 mM EDTA, 0.02% NaN3, and 10 mM MOPS, pH = 7.4) to the membrane preparation section in the presence of protease inhibitors cocktail (Calbiochem, Millipore, San Diego, CA, USA) using a glass-Teflon homogenizer before centrifugation (1000× *g*, 4 °C, 15 min). The supernatant was spun at 200,000× *g* for 15 min. The pellet (P2) was resuspended in vesicular dialysis buffer (pH 7.4), and the centrifugation was repeated. Resuspended pellet in vesicular dialysis buffer (pH 7.4) and protein concentration was determined using Pierce protein assay [33].

#### 4.7.2. Radioligand Binding Assay

The specific bindings of [^3^H]mecamylamine ([^3^H]MLA), [^3^H]cytisine, and [^3^H]quinuclidinyl benzilate ([^3^H]QNB) were used to quantify the level of α7 nAChR, heteromeric nAChR, and mAChRs, respectively [32,56,57]. A filtration-based assay was used as described in [33] to determine the reversible bindings of [^3^H]cytisine, [^3^H]MLA, and [^3^H]QNB to lung membrane fractions. [^3^H]cytisine (31.8–35.8 Ci/mmol) and [^3^H]QNB (30 Ci/mmol) were purchased from Perkin Elmer Life Science (Shelton, CT, USA). [^3^H]MLA (60 Ci/mmol) was purchased from American Radiolabeled Chemicals. Inc. (Saint Louis, MO, USA). [^3^H]cytisine and [^3^H]MLA were isotopically diluted to 8 and 12 Ci/mmol, respectively, before use. Lung membrane aliquots (100 µg protein/condition) were incubated for 60 min at room temperature with radioligands (final concentrations for [^3^H]MLA, [^3^H]QNB, and [^3^H]Cytisine were 100 nM, 1 uM, and 100 nM, respectively). Then, bound ^3^H was separated via rapid filtration through a polyethyleneimine-treated Whatman GF/B filter (GE Healthcare Life Science). Filters were immediately washed with 3–5 mL of ice-cold vesicle dialyses buffer then allowed to fully dry at room temperature before soaked with scintillation cocktail, and their ^3^H content were determined in count per minute (cpm) by liquid scintillation counting. Parallel experiments were conducted to determine the nonspecific binding in the presence of high concentrations of non-radiolabeled MLA, atropine, cytisine, for [^3^H]MLA, [^3^H]QNB, and [^3^H]cytisine binding, respectively.

### 4.8. Data Analyses

The results were analyzed with one-way ANOVA, followed by the Holm–Šidák post hoc test. Significant differences were considered when *p* < 0.05, all data are expressed as the means ± SD. Data analysis was performed through the GraphPad Prism program.

## 5. Conclusions

Our results indicate that the α7 nAChR contributes to the anti-inflammatory effects of the lung cholinergic system. The ability to increase the level of lung α7 nAChR (in WT mice) following LPS-instillation concurred with a lower level of inflammatory marker in BALF and vice versa (in VAChT-KD^HOM^ mice). In addition, in cholinergic-deficient mice unable to increase α7 nAChR level in response to LPS-instillation, treatment with selective α7 nAChR agonist restored mice’s ability to increase lung α7 nAChR and decreased the level of inflammatory marker in BALF. The increase in lung α7 nAChR in response to acute inflammation can result from upregulation of α7 nAChR expression in lung cells or by recruiting immune cells that express α7 nAChR at a high level to the lung. As such, pharmacological agents that increase the expression and/or function of lung α7 nicotinic receptors have potential clinical uses for the treatment of acute inflammatory lung conditions.

## Data Availability

The data presented in this study are available on request from the corresponding author.
